# Genetic and environmental contributions to the development of dental arch traits: a longitudinal twin study

**DOI:** 10.1093/ejo/cjaf018

**Published:** 2025-04-02

**Authors:** Jamal Giri, Michelle Bockmann, Alan Brook, Angela Gurr, Lyle Palmer, Toby Hughes

**Affiliations:** Adelaide Dental School, Faculty of Health and Medical Sciences, The University of Adelaide, 1 Frome Road, Adelaide, SA 5000, Australia; Adelaide Dental School, Faculty of Health and Medical Sciences, The University of Adelaide, 1 Frome Road, Adelaide, SA 5000, Australia; Adelaide Dental School, Faculty of Health and Medical Sciences, The University of Adelaide, 1 Frome Road, Adelaide, SA 5000, Australia; Adelaide Dental School, Faculty of Health and Medical Sciences, The University of Adelaide, 1 Frome Road, Adelaide, SA 5000, Australia; School of Public Health, Faculty of Health and Medical Sciences, The University of Adelaide, Rundle Mall Plaza, 50 Rundle Mall, Adelaide, SA 5000, Australia; Australian Institute of Machine Learning, The University of Adelaide, Corner Frome Road and North Terrace, Adelaide, SA 5000, Australia; Adelaide Dental School, Faculty of Health and Medical Sciences, The University of Adelaide, 1 Frome Road, Adelaide, SA 5000, Australia

**Keywords:** dental arch, environment, genetics, heritability, twins

## Abstract

**Objective:**

This study aimed to estimate the relative contributions of genetic and environmental factors to phenotypic variations of dental arch traits from primary to permanent dentition stages.

**Methods:**

Digital dental models of 188 Australian twin pairs (90 monozygotic and 98 dizygotic) in the primary dentition stage, followed up through the mixed and permanent dentition stages, were included in the study. Landmarks were identified on both maxillary and mandibular dental arches in MeshLab for measuring intercanine widths, intermolar widths, arch lengths, overjet, overbite and molar relationships. Genetic structural equation modelling was performed on the quantitative twin data of dental arch traits.

**Results:**

The phenotypic variance of dental arch traits was generally best explained by a model incorporating additive genetic (A) and non-shared environmental (E) components, an AE model. However, the variance of overjet in the primary dentition was best explained by shared environmental (C) and non-shared environmental (E) components. Heritability estimates were high for intra-arch traits (0.65–0.88), but low to moderate for inter-arch traits (0.21–0.51). While heritability estimates fluctuated for most traits from primary to permanent dentition stages, the estimates for arch lengths and intermolar widths were mostly above 0.8 throughout development.

**Limitation:**

Only twins of European descent were included in this study.

**Conclusions:**

Dental arch traits were mostly influenced by additive genetic and non-shared environmental factors during development. Except for arch lengths and intermolar widths, genetic and environmental influences on dental arch traits fluctuated during development, with the genetic influence at its lowest during the mixed dentition stage.

## Background

Development of the dental arches is a complex, dynamic process. Rapid changes in dental arches and occlusion occur during the primary and mixed dentition stages, before reaching the more stable permanent dentition stage, with further subtle changes occurring throughout life [1]. Several studies have investigated these changes. A significant increase in arch length and width has been reported in the first two years of life [2, 3]. Intercanine and intermolar widths increase until the early permanent dentition stage, after which they either stabilize or slightly decrease [2, 4]. During the late mixed dentition stages, mesial drift of molars decreases the arch length [5]. This also results in a change in the molar relationship, with the molar relationship in the permanent dentition strongly correlated with the molar relationship in the primary dentition [6]. Overjet and overbite usually increase during the early years of dental development and subsequently decrease during the permanent dentition stage [7]. While these anatomical changes in the dental arches are well documented, their genetic and environmental underpinnings are not well understood.

The dental arches develop as a complex adaptive system that is a component of the oro-facial complex, whose overall development is shaped by multilevel complex network interactions between genetic and environmental factors [8]. A comprehensive understanding of the development of dental arches and the factors influencing their development is crucial for clinicians to identify variations of dental arch and occlusal development. Subsequently, this enables clinicians to develop suitable treatment plans and communicate the potential changes during and after treatment to patients [3].

Studies comparing the resemblance between monozygotic (MZ) and dizygotic (DZ) twin pairs can partition the genetic and environmental influences on dental arches during development [9]. The classical twin study is the most practical design that compares MZ and DZ twins raised in the same family environment. Monozygotic twins are genetically identical at the nucleotide level, while DZ twins share on average 50% of their genes, like non-twin siblings. Utilising this difference in genetic sharing between MZ and DZ twins, relative contributions of genetic and environmental factors on dental arches and occlusion can be estimated [10].

Twin studies investigating the development of dental arches and occlusion have reported conflicting results, with findings varying from strong genetic influence [11–14] to little or negligible genetic influence [15–18]. Several of these studies relied on twin correlations to estimate heritability, rather than using robust genetic modelling to partition the relative contributions of genetic and environmental factors for specific traits. In addition, all of these studies were confined to the permanent dentition stage, except for one study each in the primary dentition [12] and the mixed dentition [18] stage.

Recent systematic reviews concluded that dental arch dimensions were under strong genetic determination, while occlusal traits demonstrated moderate to low genetic determination in the permanent dentition stage [19, 20]. However, one of the reviews particularly highlighted a need for longitudinal twin studies from primary to permanent dentition to assess genetic and environmental influences on dental arches and occlusion at various stages of development using a robust genetic analysis [19]. Therefore, using a sample of longitudinally assessed MZ and DZ twin pairs, the objectives of the current study were to:

1. Measure the changes in dental arch traits from primary to permanent dentition stages.
2. Estimate the genetic and environmental contributions to the observed phenotypic variance of the dental arch traits during development.
3. Evaluate the temporal changes in the genetic and environmental contributions from primary to permanent dentition stages.

## Materials and methods

### Study samples

Ethics approval was obtained from the Human Research Ethics Committee at the University of Adelaide (Approval number: H-2023-060) and all participants were informed volunteers. The original sample was a national cohort of 300 twin pairs of European descent without craniofacial anomalies, recruited through the National Health and Medical Research Council (NHMRC) of Australia twin registry. The twins belonged to middle-class families in Adelaide and Melbourne and were raised together. Twin zygosities were ascertained by analysing up to six highly variable genetic loci (FES, vWA31, F13A1, THO1, D21S11, FGA) on six different chromosomes from buccal cell DNA. The probability of dizygosity, given concordance for all systems was below 1%. The Craniofacial Biology Research Group at the Adelaide Dental School collected the twins’ dental records and associated metadata longitudinally between 1995 and 2006 [21]. Dental models were collected at the primary, mixed and permanent dentition stages of development. Alginate impressions of maxillary and mandibular dental arches were poured with dental stone to prepare dental models.

The stone dental models were scanned using a 3D lab scanner (3Shape, E4, Copenhagen, Denmark), and the resulting digital models were saved in Standard Tessellation Language (.stl) format. The maxillary and mandibular models were first scanned individually and then in occlusion, with a resolution of 4 µm. Digital models of the twins were included in the study at primary, mixed, and permanent dentition stages if they met the following criteria:

a. Primary dentition: Presence of all primary teeth, from central incisors to second molars.

b. Mixed dentition: Presence of primary canines and first and second primary molars, along with permanent incisors and first permanent molars.

c. Permanent dentition: Presence of all permanent teeth, from central incisors to first permanent molars.

Models were excluded if they were of poor quality, had a history of orthodontic treatment, or exhibited dental variations such as supernumerary teeth or ectopic eruptions. Based on the selection criteria, models of 188 twin pairs (90 monozygotic and 98 dizygotic pairs) were included in the primary dentition stage, while models of 181 twin pairs were included in the mixed dentition stage and 134 twin pairs in the permanent dentition stage, indicating some twins were lost to follow-up. (Table 1).

**Table 1.** Sample characteristics.

Dentition stage	Total twins	Age (Yrs)	MZ	DZ	Male	Female
Primary	376 (188 pairs)	5.8 ± 0.7	180	196	176	200
Mixed	362 (181 pairs)	9.4 ± 0.8	176	186	174	188
Permanent	268 (134 pairs)	14.2 ± 0.8	138	130	144	124

Note: MZ: Monozygotic, DZ: Dizygotic.

### Arch measurement

**Figure 1.**
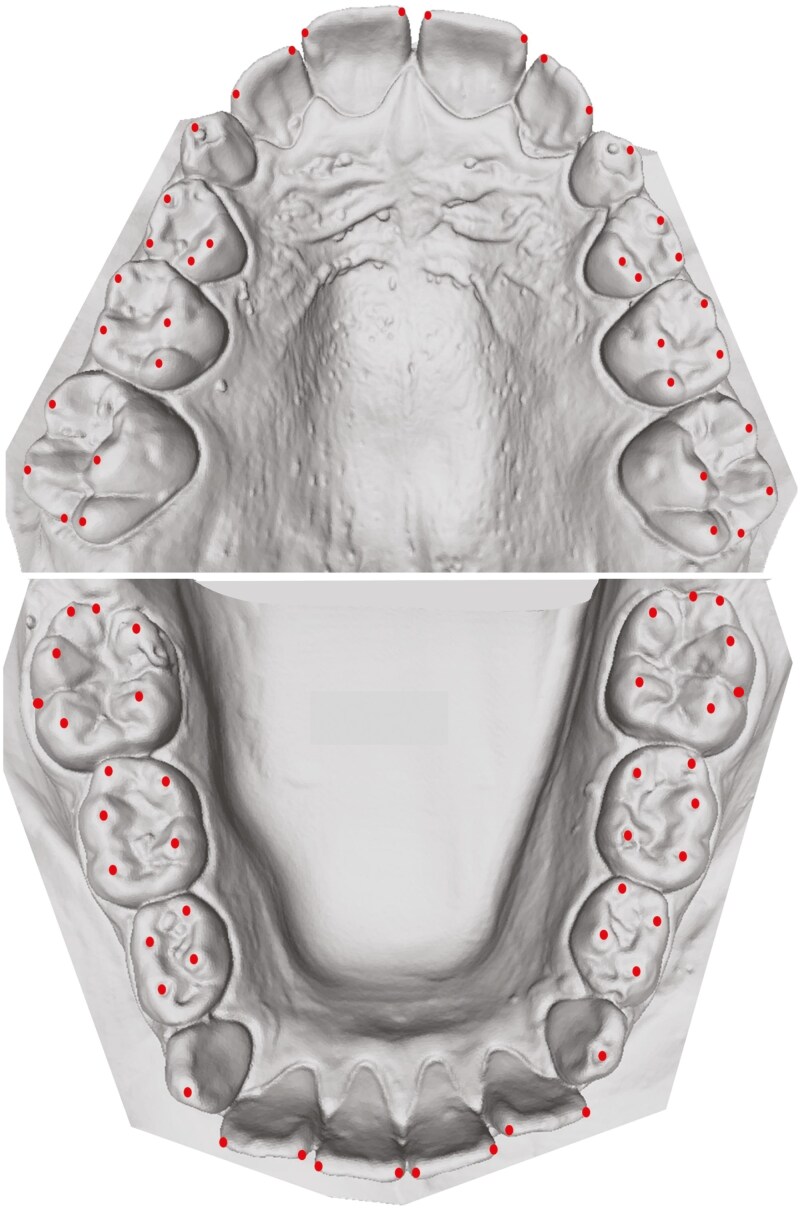
Maxillary and mandibular mixed dentition models with landmarks.

The maxillary and mandibular digital models were imported into MeshLab (ISTI-CNR, Pisa, Italy, version 2022.02) for landmark digitization, where Cartesian coordinates of each landmark were recorded. The following landmarks were digitized (Fig. 1):

a. Mesial and distal incisal edges of incisors
b. Cusp tips of canines and all posterior teeth
c. Distal contact point of first permanent molars (in permanent and mixed dentition) or distal contact point of second primary molars (in primary dentition)
d. Buccal groove of mandibular first permanent molars (in permanent and mixed dentition).

Intra-arch measurements (intercanine width, intermolar width and arch length) were calculated using the relevant digitized landmarks in both maxillary and mandibular arches and the maxillary occlusal plane was used as a reference plane for inter-arch measurements (overbite, overjet and molar relationship) (Table 2). The maxillary occlusal plane was formed by connecting the midpoint of mesial incisal points of two maxillary central incisors with the mesiobuccal cusp tips of the first permanent molars (second primary molars in primary dentition) on either side. The molar relationships were assessed on both sides of the arch, with a positive measurement indicating distocclusion and a negative measurement indicating mesiocclusion. All measurements were performed using pracma (2.3.8) [22] and geomorph (4.0.3) [23] packages in R.

**Table 2.** Definition of measured traits.

Measurement	Traits	Definitions
Intra-arch	Intercanine width	**Primary and mixed dentitions:** Distance between the cusp tips of right and left primary canines **Permanent dentition:** Distance between the cusp tips of right and left permanent canines
	Intermolar width	**Primary dentition:** Distance between the mesiobuccal cusp tips of right and left primary second molars **Permanent and mixed dentitions:** Distance between the mesiobuccal cusp tips of right and left permanent first molars
	Arch length	**Primary dentition:** Perpendicular distance between the midpoint of mesial incisal points of two central incisors and the line joining the distal contact points of right and left primary second molars **Permanent and mixed dentition:** Perpendicular distance between the midpoint of mesial incisal points of two central incisors and the line joining the distal contact points of right and left permanent first molars
Inter-arch	Overjet	Horizontal overlap between maxillary and mandibular incisors
	Overbite	Vertical overlap between maxillary and mandibular incisors
	Molar relationship (right and left)	**Primary dentition:** Relative position of distal contact points of maxillary and mandibular primary second molars in antero-posterior direction measured in millimetres. **Permanent and mixed dentition:** Relative position of mesiobuccal cusp tip of the maxillary first permanent molar and the buccal groove of the mandibular first permanent molar in antero-posterior direction measured in millimetres.

### Reliability analysis

All digital models were landmarked by a single examiner (JG). To assess intra-examiner reliability, the same examiner repeated landmark identification on a randomly selected subset of 60 models (20 from each dentition stage: primary, mixed, and permanent) after four weeks. A second examiner (AG) identified landmarks on the same 60 models to evaluate inter-examiner reliability. Systematic error was computed using the two-way mixed effects, absolute agreement, and single measurement type intraclass correlation coefficient (ICC), while random error was estimated using Dahlberg’s formula [24].

### Statistical analysis

The Shapiro-Wilk test was used to determine the normality of intra- and inter-arch measurements and frank outliers were identified using Z-score analysis. Means and standard deviations of arch measurements were calculated. To evaluate the influence of sex, zygosity, dentition stage and family membership on arch measurements, linear mixed-effects models were developed [25]. The models included sex, zygosity and dentition stage as fixed effects, with family ID modelled as a random effect to capture the within-family clustering of twin pairs. Independent samples t-tests were used to compare the dental arch trait means during the mixed dentition stage between twins who were lost to follow-up and those who continued to the permanent dentition stage. Statistical analyses were performed in R (version 4.3.2). Statistical significance was set at p < .05.

### Genetic analysis

**Figure 2.**
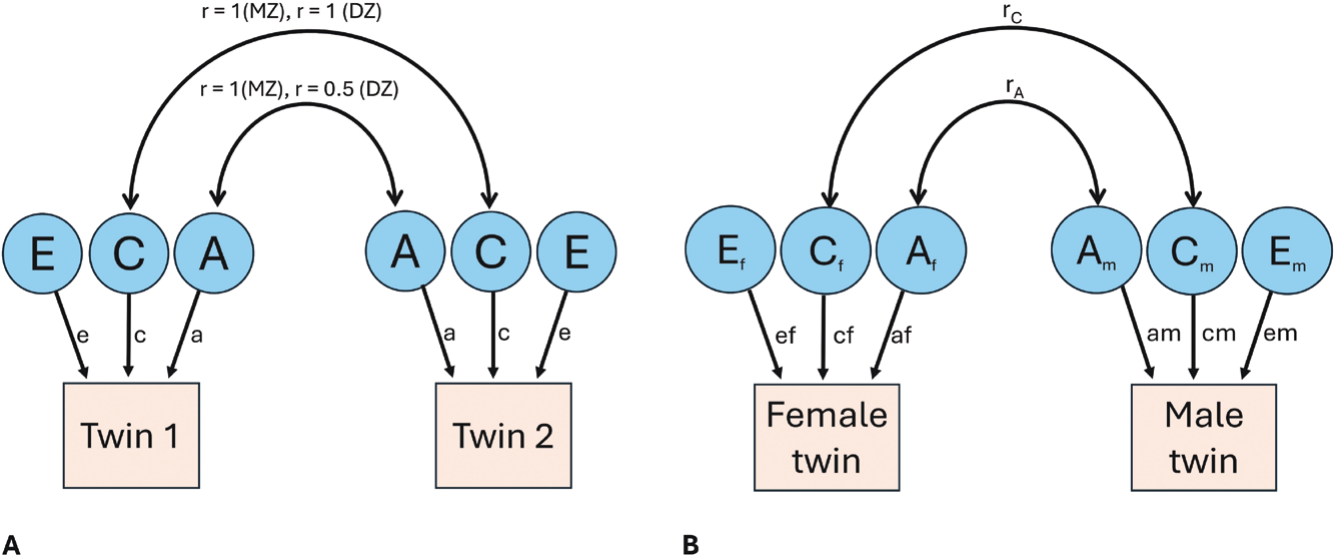
Path diagrams showing three potential factors influencing twin pairs’ phenotypes: additive genetic factors (A), shared environmental factors (C), and nonshared environmental factors (E). **A. ACE model**: Path coefficients (a, c and e) denote the relative contributions of each factor, while double arrowheads represent the correlations (r) between latent factors among twin pairs. **B. Full sex-limitation model showing opposite-sex twin pairs:** A, C and E variances are estimated separately for females (f) and males (m). Paths af, cf, and ef denote the A, C, and E effects on the trait for females, while am, cm, and em represent these effects for males. rC and rA represent estimated shared environmental and additive genetic correlations among twin pairs.

For each dental arch trait, ICCs were calculated between MZ and DZ twin pairs. To determine the genetic and environmental influences on dental arch traits at each stage of dental development, genetic structural equation modelling (SEM) was performed on the twin data using the OpenMx package in R [26] (Fig. 2). The following sources of genetic and environmental variations were considered in the model:

1. Additive genetic factors (A): genetic factors that contribute to a phenotype through cumulative actions of individual genes.
2. Non-additive genetic factors (D): genetic factors that contribute to a phenotype through gene interactions, such as dominance and epistasis.
3. Shared environmental factors (C): common environmental factors shared by both twins raised in the same family environment that influence a phenotype.
4. Non-shared environmental factors (E): environmental factors unique to each twin that influence a phenotype.

The model operated under the assumptions of the classical twin method (i.e. random mating, equal trait-related shared environmental influences on both MZ and DZ twins, and the absence of gene-environment (GE) covariation or GE interaction) [27]. Implicit in the genetic model is an expected pattern of correlations that illustrates how various factors influence MZ and DZ twins. Additive genetic (A) influences show a perfect correlation (1.0) for MZ twins and 0.5 for DZ twins, while non-additive genetic (D) influences yield correlations of 1.0 for MZ twins and 0.25 for DZ twins. Shared environmental (C) influences are expected to correlate perfectly (1.0) for both MZ and DZ twins raised together, whereas non-shared environmental (E) influences are uncorrelated between twins [28].

ACE and ADE models were fitted to the dental arch data of the twins to estimate the contribution of additive genetic (A), non-additive genetic (D), shared environmental (C) and non-shared environmental (E) factors to the total phenotypic variance. These models were fitted separately because the C and D variance components are confounded and cannot be estimated simultaneously in twins raised together [29].

Twin data was fit to a full sex-limitation model to account for both qualitative and quantitative sex differences, with sub-models including a quantitative sex differences model and an ACE or ADE model without sex differences. Chi-square likelihood ratio tests were used to compare the goodness-of-fit between more complex models and their corresponding sub-models (e.g. a full sex-limitation model vs. ACE model without sex differences). A more complex model was accepted only if a simpler model showed a significant loss of fit (p < .05) according to the principle of parsimony. Akaike’s information criteria (AIC) was used to evaluate the fit of non-nested models (e.g. CE vs. AE) with a smaller value suggesting a better fit. Narrow-sense heritability (h^2^) estimates were calculated for each dental arch trait as the ratio of additive genetic variation to total phenotypic variation for the best-fitting model.

## Results

### Reliability analysis

The ICCs showed high intra-examiner (0.97–0.99) and inter-examiner (0.95–0.99) reliability. Dahlberg’s errors were below 0.5 mm for intra-examiner (0.10–0.33 mm) and inter-examiner (0.18–0.43 mm) measurements (Supplementary Table 1). Overall, measurement errors were small and unlikely to bias the study results.

### Descriptive statistics

No statistically significant differences were observed between the MZ and the DZ twins’ dental arch measurements in all three stages of development. However, sexual dimorphism was evident in most intra-arch measurements, with males consistently exhibiting larger values than females across all dentition stages. These differences remained statistically significant after applying the Bonferroni correction to the overall significance level of 0.05 (Table 3). No statistically significant differences were found in the dental arch trait means during the mixed dentition stage between twins who were lost to follow-up and those who continued to the permanent dentition stage. This indicates that attrition of the sample likely did not bias the results.

**Table 3.** Mean values of intra- and inter-arch traits in different stages of dentition.

Arch measurements in different stages of dentition	Mean (SD)					p-value	
	Overall	MZ	DZ	Male	Female	MZ vs. DZ	Male vs. Female
**Primary**							
Maxillary intercanine width	28.5 (2.1)	28.7 (1.9)	28.3 (2.1)	29.1 (1.9)	28.0 (2.0)	0.092	<0.001*
Maxillary intermolar width	40.9 (2.3)	41.1 (2.3)	40.8 (2.2)	41.7 (2.2)	40.2 (2.1)	0.249	<0.001*
Maxillary arch length	25.9 (1.7)	25.9 (1.7)	25.8 (1.7)	26.2 (1.5)	25.6 (1.8)	0.607	0.001*
Mandibular intercanine width	22.8 (1.9)	23.1 (2.1)	22.7 (1.9)	23.2 (2.1)	22.5 (1.8)	0.186	0.002*
Mandibular intermolar width	35.4 (2.0)	35.5 (2.1)	35.2 (1.9)	35.9 (2.1)	34.9 (1.8)	0.147	<0.001*
Mandibular arch length	23.6 (1.5)	23.6 (1.5)	23.6 (1.6)	23.9 (1.3)	23.3 (1.6)	0.828	<0.001*
Overbite	1.1 (0.7)	1.1 (0.8)	1.1 (0.7)	1.1 (0.8)	1.0 (0.7)	0.771	0.319
Overjet	2.4 (1.2)	2.3 (1.1)	2.4 (1.2)	2.3 (0.9)	2.4 (1.4)	0.830	0.537
Right molar relationship	−0.03 (1.1)	−0.04 (1.1)	−0.02 (1.1)	0.04 (1.2)	−0.10 (1.1)	0.840	0.244
Left molar relationship	−0.02 (1.1)	0.01 (1.1)	−0.07 (1.0)	0.03 (1.1)	−0.07 (1.1)	0.335	0.349
**Mixed**							
Maxillary intercanine width	31.3 (3.5)	31.1 (3.4)	31.4 (3.5)	31.8 (3.2)	30.7 (3.6)	0.401	0.004
Maxillary intermolar width	49.1 (2.7)	49.2 (2.8)	48.9 (2.6)	49.8 (2.7)	48.4 (2.5)	0.263	<0.001*
Maxillary arch length	37.5 (2.2)	37.5 (2.1)	37.5 (2.2)	38.1 (2.0)	36.9 (2.1)	0.956	<0.001*
Mandibular intercanine width	25.2 (2.7)	25.1 (2.6)	25.3 (2.8)	25.7 (2.5)	24.7 (2.7)	0.274	<0.001*
Mandibular intermolar width	43.7 (2.4)	44.1 (2.4)	43.5 (2.3)	44.5 (2.3)	43.1 (2.2)	0.051	<0.001*
Mandibular arch length	34.3 (1.9)	34.3 (1.8)	34.3 (1.9)	34.8 (1.6)	33.8 (1.9)	0.770	<0.001*
Overbite	1.7 (1.1)	1.7 (1.1)	1.8 (1.0)	1.9 (1.2)	1.6 (0.9)	0.169	0.004
Overjet	3.2 (1.2)	3.2 (1.3)	3.2 (1.1)	3.4 (1.2)	3.0 (1.1)	0.996	0.012
Right molar relationship	1.7 (1.4)	1.8 (1.5)	1.7 (1.3)	1.8 (1.4)	1.7 (1.4)	0.181	0.297
Left molar relationship	1.6 (1.4)	1.5 (1.4)	1.6 (1.3)	1.6 (1.5)	1.6 (1.3)	0.599	0.991
**Permanent**							
Maxillary intercanine width	33.1 (3.1)	32.9 (2.9)	33.1 (3.3)	33.7 (2.6)	32.2 (3.4)	0.650	<0.001*
Maxillary intermolar width	50.5 (3.2)	50.6 (3.3)	50.5 (3.1)	51.3 (3.2)	49.6 (3.0)	0.715	<0.001*
Maxillary arch length	36.5 (2.5)	36.4 (2.4)	36.6 (2.6)	37.3 (2.3)	35.5 (2.4)	0.683	<0.001*
Mandibular intercanine width	25.3 (1.9)	25.4 (1.8)	25.1 (2.0)	25.5 (2.0)	25.0 (1.8)	0.211	0.024
Mandibular intermolar width	44.1 (2.8)	44.1 (3.0)	43.9 (2.7)	44.9 (2.9)	43.1 (2.4)	0.627	<0.001*
Mandibular arch length	32.8 (2.3)	32.6 (2.2)	32.9 (2.5)	33.5 (2.1)	31.9 (2.3)	0.282	<0.001*
Overbite	2.2 (1.1)	2.2 (1.2)	2.2 (1.1)	2.4 (1.2)	2.0 (1.0)	0.880	0.014
Overjet	3.2 (1.1)	3.3 (1.1)	3.2 (1.1)	3.4 (1.0)	3.1 (1.0)	0.719	0.019
Right molar relationship	0.6 (1.7)	0.6 (1.8)	0.6 (1.7)	0.6 (1.7)	0.6 (1.7)	0.856	0.948
Left molar relationship	0.5 (1.6)	0.5 (1.7)	0.5 (1.6)	0.6 (1.7)	0.4 (1.6)	0.794	0.154

Note: MZ: Monozygotic, DZ: Dizygotic, SD: Standard deviation, *: statistically significant difference at p < .0025 after Bonferroni correction.

### Genetic analysis

**Figure 3.**
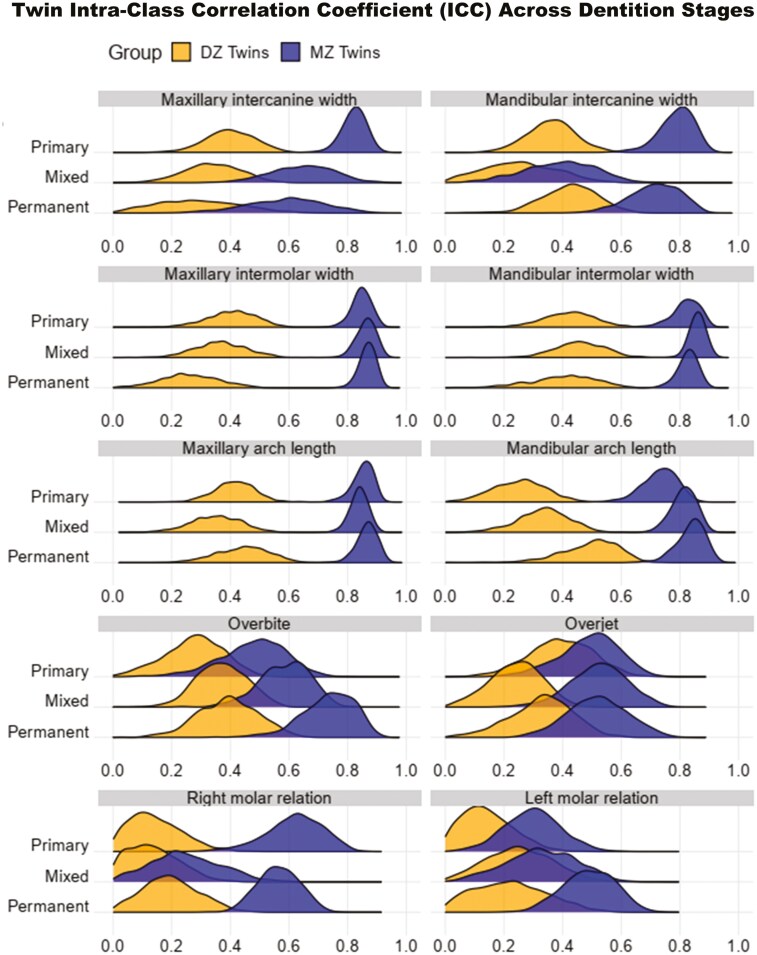
Ridge plots with density curves for twin intra-class correlation coefficient (ICC) distribution.

For intra-arch measurements, ICCs ranged from 0.40–0.87 for MZ twins and 0.25–0.50 for DZ twins. However, for inter-arch measurements, the ICCs ranged from 0.23–0.74 for MZ twins and 0.11–0.40 for DZ twins. Ridge plots (Fig. 3) depict the bootstrapped distributions of ICCs between MZ and DZ twins for intra-arch and inter-arch measurements across different dentition stages. For the intercanine widths in maxillary and mandibular arches, the distribution of ICCs between MZ and DZ twins changed from the primary to the permanent dentition, resulting in increased overlap. There were considerable overlaps in the distribution of ICCs between MZ and DZ twins for overbite, overjet, right molar relationship and left molar relationship from primary to permanent dentition stages. However, the distributions of ICCs between MZ and DZ twins for intermolar widths and arch lengths for both arches were relatively stable across the dentition stages with clear separations between the distributions.

A model with additive genetic and non-shared environmental variance components (AE model) best explained the phenotypic variations for all dental arch traits, except overjet in the primary dentition stage, for which the shared and non-shared environmental variance components (CE model) better explained the phenotypic variation (Supplementary Table 2). Heritability estimates were calculated from pooled male and female data for most traits. However, significant heterogeneity for the variance components was detected between the sexes for maxillary intercanine width in the permanent dentition and overjet in the primary dentition, so the heritability estimates were reported separately for males and females. For maxillary intercanine width in the permanent dentition, females demonstrated a higher additive genetic variance than males (0.65 vs. 0.58 respectively).

Overall, narrow-sense heritability estimates for intra-arch traits were high across primary to permanent dentition, ranging between 0.65–0.88, except for the mandibular intercanine width in the mixed dentition (0.45). Narrow-sense heritability estimates for inter-arch traits were low to moderate, with values ranging between 0.21–0.51 except for the overbite where the estimates were between 0.56–0.72 (Table 4).

**Table 4.** Estimates of variance components and 95% confidence intervals for intra- and inter-arch traits in different stages of dentition.

Dental arch traits in different stages of dentition	Model	Variance components						
		A	95% CI	C	95% CI	E	95% CI	h^2^
**Primary**								
Maxillary intercanine width	AE	0.85	0.78–0.89			0.15	0.10–0.21	0.85
Maxillary intermolar width	AE	0.85	0.78–0.88			0.15	0.11–0.21	0.85
Maxillary arch length	AE	0.86	0.79–0.89			0.14	0.10–0.20	0.86
Mandibular intercanine width	AE	0.75	0.65–0.81			0.25	0.18–0.34	0.75
Mandibular intermolar width	AE	0.82	0.74–0.86			0.18	0.13–0.25	0.82
Mandibular arch length	AE	0.74	0.62–0.82			0.26	0.17–0.37	0.74
Overbite	AE	0.60	0.44–0.71			0.40	0.28–0.55	0.60
Overjet^#^	CE			0.39 0.57	0.17–0.57 0.32–0.72	0.61 0.43	0.42–0.82 0.27–0.67	
Right molar relationship	AE	0.40	0.25–0.52			0.60	0.47–0.74	0.40
Left molar relationship	AE	0.27	0.09–0.43			0.73	0.56–0.90	0.27
**Mixed**								
Maxillary intercanine width	AE	0.65	0.52–0.74			0.35	0.25–0.47	0.65
Maxillary intermolar width	AE	0.85	0.79–0.89			0.15	0.10–0.20	0.85
Maxillary arch length	AE	0.85	0.78–0.89			0.15	0.10–0.21	0.85
Mandibular intercanine width	AE	0.45	0.25–0.60			0.55	0.39–0.74	0.45
Mandibular intermolar width	AE	0.85	0.79–0.89			0.15	0.11–0.20	0.85
Mandibular arch length	AE	0.83	0.75–0.88			0.17	0.11–0.24	0.83
Overbite	AE	0.56	0.42–0.66			0.44	0.33–0.57	0.56
Overjet	AE	0.51	0.35–0.62			0.49	0.37–0.64	0.51
Right molar relationship	AE	0.21	0.03–0.37			0.79	0.62–0.96	0.21
Left molar relationship	AE	0.27	0.10–0.42			0.73	0.57–0.90	0.27
**Permanent**								
Maxillary intercanine width^#^	AE	0.65 0.58	0.42–0.78 0.22–0.78			0.35 0.42	0.21–0.57 0.21–0.77	0.65 0.58
Maxillary intermolar width	AE	0.86	0.78–0.90			0.14	0.10–0.21	0.86
Maxillary arch length	AE	0.88	0.81–0.91			0.12	0.08–0.18	0.88
Mandibular intercanine width	AE	0.74	0.62–0.81			0.26	0.18–0.37	0.74
Mandibular intermolar width	AE	0.81	0.72–0.86			0.19	0.13–0.27	0.81
Mandibular arch length	AE	0.86	0.79–0.90			0.14	0.10–0.21	0.86
Overbite	AE	0.72	0.61–0.80			0.28	0.19–0.38	0.72
Overjet	AE	0.53	0.36–0.66			0.47	0.33–0.63	0.53
Right molar relationship	AE	0.54	0.36–0.67			0.46	0.32–0.63	0.54
Left molar relationship	AE	0.45	0.27–0.60			0.55	0.39–0.72	0.45

Note: additive genetic variance: (A), shared environmental variance: (C), non-shared environmental variance: (E), confidence interval: (CI), narrow-sense heritability: (h^2^), ^#^: female and male values for traits with significant heterogeneity between sexes.

## Discussion

The current study investigated the development of dental arch traits from the primary to permanent dentition stages to determine the relative contributions of genetic and environmental factors during development using SEM. Based on the findings, a model including additive genetic and non-shared environmental components (AE model) best explained the observed phenotypic variations for all dental arch traits, except overjet in the primary dentition, which was under shared and non-shared environmental influences (CE model).

To our knowledge, this is the first longitudinal twin study quantifying the genetic and environmental influences on dental arches and occlusion during development from primary to permanent dentition stages. The longitudinal nature of the study enabled us to assess the change in the genetic influence over time. Heritability is known to be time-dependent because it can fluctuate with changes in genetic variance and environmental factors [30]. The ridge plots (Fig. 3) illustrate variations in genetic influences on dental arch traits, as seen in the changes in the overlap or separation of ICC distributions between MZ and DZ twins from primary to permanent dentition. These observations are further supported by heritability estimates obtained from genetic modelling. For arch lengths and intermolar widths in maxillary and mandibular arches, heritability estimates were mostly above 0.8 throughout development indicating a strong genetic influence. However, the heritability estimates of intercanine width, which were lower than that of intermolar widths, fluctuated during development suggesting a greater environmental influence in the anterior region of dental arches. This could be because functional activities have a greater influence in the anterior region of the arches and the smaller roots of anterior teeth make them more susceptible to displacement [13]. The inter-arch traits (overbite, overjet and molar relationship) displayed low to moderate heritability estimates except for the estimate for overbite in permanent dentition, which was high. While heritability estimates for inter-arch traits were low to moderate, their values fluctuated, increasing slightly from the primary to the permanent dentition stages. In general, the intra-arch traits displayed high heritability while the inter-arch traits had moderate to low heritability during development.

For dental arch traits whose heritability estimates fluctuated, the lowest heritability estimates were observed in the mixed dentition stage. Additive genetic variances for both maxillary and mandibular intercanine widths dropped considerably from the primary to the mixed dentition stage. This could be attributed to the mixed dentition stage being a dynamic period with the transition of teeth and dentoalveolar growth, making it more susceptible to environmental influences [31]. Deleterious habits such as tongue thrusting and non-nutritive sucking could also influence phenotypic variances of dental arch traits during this stage. While the additive genetic variance for mandibular intercanine width rebounded from 0.45 in the mixed dentition to 0.74 in the permanent dentition, it remained relatively stable for maxillary intercanine width across these stages. This suggests that mandibular intercanine width is under strong genetic determination in the permanent dentition and should be preserved during orthodontic treatment. Our findings that heritability estimates were lowest during the mixed dentition stage contradict those of Harris and Johnson [32], who reported a median heritability estimate of 0.5 for dental arch traits in the primary dentition, which gradually diminished to near-zero values in the permanent dentition. Caution is advised while interpreting their findings as they [31] relied on a crude method of estimating heritability by doubling sibling correlation on a relatively small sample size of 45 North American siblings who were not twins.

Statistically significant phenotypic differences were observed between male and female twins for several dental arch traits. To determine whether there are different sets of genetic or environmental factors between males and females, full sex limitation models were fit to the data. The inclusion of dizygotic opposite-sex twins allowed for the estimation of both qualitative and quantitative sex differences [33]. However, no qualitative sex differences were observed in the dental arch traits in our study. While all intra-arch traits exhibited statistically significant phenotypic differences between males and females, sex differences in genetic and environmental influences were only found in maxillary intercanine width in the permanent dentition, indicating that genetic and environmental effects were largely similar for most dental arch traits for both sexes. Surprisingly, while overjet in the primary dentition showed no significant phenotypic difference between males and females, sex differences were detected in the underlying environmental influences. This finding warrants further investigation.

Several studies have explored the genetic influence on dental arches in the permanent dentition stage [13–17, 34, 35]. However, most earlier studies [15–17, 34, 35] have relied on heritability estimates derived solely from trait correlations among twins, often reporting a wide range of values resulting in estimates that were negative or greater than one. This approach is not robust enough to partition the different sources of variation into genetic and environmental factors. Using genetic SEM, we precisely estimated narrow-sense heritability and partitioned the variance of dental arch traits into additive genetic, shared environmental and non-shared environmental components in our sample.

Our finding that a model including additive genetic and non-shared environmental components best explained the sources of variation for intra-arch traits aligns with Eguchi et al. [13] and Lin et al. [14], except for mandibular intercanine width where they found the CE model as the most parsimonious. The finding of Eguchi et al [13] that the CE and AE models best described the observed variation in the mandibular intercanine width in females and males respectively is particularly interesting. This suggests that the sources of variation for mandibular intercanine width differ between males and females, with greater environmental influence observed in females. Overall, heritability estimates for intra-arch traits were comparable to those reported by Eguchi et al. [13] and Lin et al. [14], who also studied Australian twins of European ancestry and found relatively high heritability values.

For overbite and overjet our findings were similar to Lin et al. [14], both in terms of the best fitting model (AE) and estimates of heritability, though our heritability estimate for overbite was slightly higher. However, our findings differed from those of Sidlauskas et al. [36], who reported that variances in overjet and overbite were best explained by the CE and DE models, respectively. While Sidlauskas et al. [36] measured overjet and overbite using lateral cephalograms instead of dental models, this methodological difference does not fully account for the discrepancy, particularly regarding the DE model. Dominant genetic influence without any additive genetic contribution is biologically implausible [37]; therefore, this finding warrants further investigation.

Genetic and environmental influences on dental arches during the mixed dentition stage have not been investigated using genetic SEM. While Chaaban et al. [18] reported extremely low (near zero) heritability estimates for maxillary intercanine and intermolar widths in the mixed dentition using twin correlations among North American twins, these findings should be interpreted with caution. The authors [18] have acknowledged that their method of estimating heritability produced some spurious values. A decrease in heritability estimates for maxillary and mandibular intercanine widths during the mixed dentition stage was seen in our samples. Although, our estimates were of modest heritability during this stage, they were not as low as those reported by Chaaban et al. [18]. Further studies examining the genetic and environmental influences on dental arches during the mixed dentition stage will be important, as this stage is considered a critical period for preventive and interceptive orthodontics [38, 39]. Further, a reduced genetic or greater environmental influence during this stage may indicate an optimal time for orthodontic intervention.

Our findings largely align with those of Hughes et al. [12], the only twin study in primary dentition using a genetic SEM. They found that the additive genetic and non-shared environmental factors explained the phenotypic variances for arch widths, arch length, overjet and overbite. However, for overjet, our study found that shared and non-shared environmental factors best explained the phenotypic variance. The use of pacifiers along with other environmental factors during the primary dentition stage could influence overjet. However, it cannot be stated with certainty whether pacifiers were used by our twins. Our findings relating to high heritability estimates for all intra-arch traits and a moderate heritability for overbite are consistent with the findings of Hughes et al. [12]. An unusual finding in the primary dentition was the difference in genetic and environmental influences on right and left molar relationships. Although both were strongly influenced by non-shared environmental factors, there was a discrepancy of 0.13 in additive genetic variance between the right and left sides. However, the lack of similar studies in primary dentition limits direct comparison. It can be hypothesized that non-shared environmental factors might contribute to these differences by exerting asymmetric functional forces on the developing dental arches. These factors include a preference for chewing on one side, digit sucking, and unilateral tongue posture.

From a clinical perspective, a comprehensive understanding of genetic and environmental influences on dental arches during development is crucial for orthodontists to effectively manage various malocclusions. Each malocclusion occupies a unique position on the genetic-environmental spectrum, with the relative contributions of genetic and environmental factors significantly influencing orthodontic outcomes [40]. Malocclusions linked to dental arch traits with strong environmental influences are likely to respond effectively to orthodontic interventions while malocclusions associated with traits under strong genetic influences may be more demanding to treat. This is also crucial for the stability of treatment outcomes, as stability hinges on establishing a new balance between genetic and environmental factors [41]. Since the genetic and environmental influences on some dental arch traits tend to fluctuate, timing the intervention when the environmental influence is greater could lead to more favourable orthodontic outcomes. A better understanding of the fluctuating genetic influences on dental arches could enable orthodontists to plan preventive and interceptive interventions to address unfavourable development of dental arches and occlusion. In addition, this will assist orthodontists in identifying the limitations of orthodontic treatment.

Key strengths of this study include its longitudinal design, accurate ascertainment of twin zygosity and the use of robust genetic analyses to partition the relative contributions of genetic and environmental influences on dental arches. However, a limitation is the inclusion of twins of European descent only, which limits the generalizability of the findings to other populations. Another limitation of this study is the exclusive use of digital dental models, which cannot capture functional changes that influence dental arch development. Findings in the permanent dentition should be interpreted considering the attrition in sample size from the primary to the permanent dentition.

More longitudinal twin and family studies assessing the changes in dental arches from the primary to the permanent dentition stages are needed to enhance our understanding of the aetiology of malocclusion. Future studies should evaluate the genetic and environmental influences on intra- and inter-arch traits during development in different populations because population-specific factors influence heritability. Studies should also investigate the role of genetic and environmental factors in the development of dental arch shape using the geometric morphometric approach.

## Conclusions

1. All arch dimensions increased from the primary to the permanent dentition stages, except for arch length in both the maxillary and mandibular arch, which decreased between the mixed and permanent dentition stages.
2. The additive genetic and non-shared environmental factors mostly influenced the development of dental arches from the primary to permanent dentition stages. Intra-arch traits, particularly arch length and intermolar width in both arches, exhibited a strong genetic influence while the genetic influence on inter-arch traits was moderate to low. Overall, dental arches of males and females were influenced by similar genetic and environmental factors.
3. The genetic and environmental influences on dental arch traits fluctuated during development from the primary to the permanent dentition stages except for arch lengths and intermolar widths. The genetic influence was at its lowest during the mixed dentition stage for arch traits with fluctuating genetic influence.

## Supplementary Material

cjaf018_suppl_Supplementary_Tables_1

cjaf018_suppl_Supplementary_Tables_2

## Data Availability

The data will be made available upon reasonable request to the corresponding author.
