# Supplementary material for: Genetic and environmental contributions to the development of dental arch traits: a longitudinal twin study
Source: Eur J Orthod. 2025 Apr 2;47(2):cjaf018. doi: 10.1093/ejo/cjaf018 (PMC11961300; doi:10.1093/ejo/cjaf018)
Supplement: cjaf018_suppl_Supplementary_Tables_1 [file cjaf018_suppl_supplementary_tables_1.docx]

| **Dental arch traits** | **ICC**  **Intra-examiner** | **ICC**  **Inter-examiner** | **Dahlberg’s error Intra-examiner** | **Dahlberg’s error**  **Inter-examiner** |
| --- | --- | --- | --- | --- |
| **Maxillary intercanine width** | 0.99 | 0.98 | 0.31 mm | 0.43 mm |
| **Maxillary intermolar width** | 0.99 | 0.99 | 0.28 mm | 0.35 mm |
| **Maxillary arch length** | 0.99 | 0.99 | 0.21 mm | 0.31 mm |
| **Mandibular intercanine width** | 0.97 | 0.98 | 0.33 mm | 0.28 mm |
| **Mandibular intermolar width** | 0.99 | 0.99 | 0.32 mm | 0.43 mm |
| **Mandibular arch length** | 0.99 | 0.99 | 0.21 mm | 0.18 mm |
| **Overbite** | 0.99 | 0.95 | 0.10 mm | 0.26 mm |
| **Overjet** | 0.98 | 0.96 | 0.13 mm | 0.22 mm |
| **Right molar relationship** | 0.96 | 0.94 | 0.28 mm | 0.29 mm |
| **Left molar relationship** | 0.97 | 0.96 | 0.27 mm | 0.33 mm |

**Supplementary Table 1: Systematic and random errors for dental arch trait measurements**

ICC: Intra-Class Correlation Coefficient
