# Supplementary material for: Genetic and environmental contributions to the development of dental arch traits: a longitudinal twin study
Source: Eur J Orthod. 2025 Apr 2;47(2):cjaf018. doi: 10.1093/ejo/cjaf018 (PMC11961300; doi:10.1093/ejo/cjaf018)
Supplement: cjaf018_suppl_Supplementary_Tables_2 [file cjaf018_suppl_supplementary_tables_2.docx]

**Supplementary Table 2: AIC values of the models**

| **Dental arch traits** | **ADE** | **ACE** | **ACErc** | **ACEq** | **AE** | **CE** |
| --- | --- | --- | --- | --- | --- | --- |
| **Primary** |  |  |  |  |  |  |
| Maxillary intercanine width | 1496.6 | 1476.5 | 1483.6 | 1481.8 | **1476.2** | 1515.7 |
| Maxillary intermolar width | 1575.8 | 1544.7 | 1546.6 | 1545.8 | **1543.8** | 1576.7 |
| Maxillary arch length | 1260.9 | 1251.1 | 1254.8 | 1252.8 | **1249.1** | 1285.0 |
| Mandibular intercanine width | 1499.6 | 1490.1 | 1495.6 | 1493.7 | **1488.1** | 1511.7 |
| Mandibular intermolar width | 1481.6 | 1468.5 | 1471.1 | 1469.3 | **1467.2** | 1493.2 |
| Mandibular arch length | 1237.8 | 1221.9 | 1232.4 | 1230.5 | **1221.8** | 1245.3 |
| Overbite | 651.5 | 643.3 | 650.5 | 648.5 | **641.7** | 642.9 |
| Overjet | 898.5 | 896.2 | 896.3 | 894.2 | 893.5 | **891.0^#^** |
| Right molar relationship | 1078.4 | 1055.8 | 1059.5 | 1057.6 | **1053.8** | 1064.3 |
| Left molar relationship | 1040.8 | 1036.1 | 1043.6 | 1041.7 | **1034.1** | 1034.5 |
| **Mixed** |  |  |  |  |  |  |
| Maxillary intercanine width | 1834.3 | 1828.5 | 1832.7 | 1830.8 | **1826.8** | 1833.9 |
| Maxillary intermolar width | 1624.7 | 1602.2 | 1604.8 | 1603.1 | **1600.3** | 1633.9 |
| Maxillary arch length | 1476.2 | 1459.8 | 1463.9 | 1462.1 | **1457.8** | 1499.2 |
| Mandibular intercanine width | 1647.5 | 1633.3 | 1640.8 | 1638.2 | **1635.8*** | 1638.9 |
| Mandibular intermolar width | 1518.2 | 1490.8 | 1495.9 | 1493.9 | **1490.6** | 1513.9 |
| Mandibular arch length | 1352.4 | 1333.8 | 1337.2 | 1335.3 | **1331.8** | 1365.3 |
| Overbite | 1047.8 | 1039.6 | 1041.6 | 1039.8 | **1038.6** | 1041.5 |
| Overjet | 1086.1 | 1077.1 | 1082.6 | 1082.1 | **1075.9** | 1081.1 |
| Right molar relationship | 1271.2 | 1264.1 | 1268.7 | 1266.0 | **1264.3*** | 1264.8 |
| Left molar relationship | 1242.6 | 1237.4 | 1238.2 | 1236.3 | **1236.2** | 1236.5 |
| **Permanent** |  |  |  |  |  |  |
| Maxillary intercanine width | 1281.9 | 1267.0 | 1263.9 | 1262.2 | **1255.3^#^** | 1258.8 |
| Maxillary intermolar width | 1294.6 | 1287.5 | 1292.7 | 1290.8 | **1285.1** | 1319.8 |
| Maxillary arch length | 1157.5 | 1136.5 | 1143.2 | 1121.3 | **1135.0** | 1161.1 |
| Mandibular intercanine width | 1064.3 | 1062.9 | 1066.7 | 1064.8 | **1062.2** | 1075.2 |
| Mandibular intermolar width | 1242.2 | 1224.8 | 1227.5 | 1225.6 | **1224.2** | 1238.4 |
| Mandibular arch length | 1125.4 | 1108.7 | 1109.6 | 1109.2 | **1107.8** | 1127.1 |
| Overbite | 798.4 | 794.9 | 796.5 | 794.3 | **793.2** | 801.7 |
| Overjet | 796.9 | 779.1 | 786.4 | 783.9 | **777.3** | 779.5 |
| Right molar relationship | 1060.8 | 1046.2 | 1052.7 | 1050.9 | **1044.3** | 1050.9 |
| Left molar relationship | 1038.4 | 1028.4 | 1031.0 | 1029.4 | **1028.4** | 1031.5 |

Note: ADE - Additive genetic, non-additive genetic and non-shared environment model; ACE - Additive genetic, shared environment and non-shared environment model; ACErc - Qualitative & quantitative sex differences ACE model; ACEq - Quantitative non-scalar sex differences ACE model; AE - Additive genetic and non-shared environment model; CE - Shared environment and non-shared environment model; Values in bold - best-fit models; # - Quantitative sex differences observed between males and females (model is nested within the ACEq model); * - Simpler model (AE), nested within the ACE model selected based on Chi-square likelihood ratio test (p < 0.05).
